# Dr. Alonzo T. Smith

**Published:** 1901-04

**Authors:** 


Dr. Alonzo T. Smith, of Syracuse, died March 8, 1901, aged 81 years. He had been a prominent physician in that city and was regarded as the oldest alumnus of Geneva Medical College.

